# Structure and biochemical properties of recombinant human dimethylglycine dehydrogenase and comparison to the disease‐related H109R variant

**DOI:** 10.1111/febs.13828

**Published:** 2016-10-06

**Authors:** Peter Augustin, Altijana Hromic, Tea Pavkov‐Keller, Karl Gruber, Peter Macheroux

**Affiliations:** ^1^ Institute of BiochemistryGraz University of TechnologyAustria; ^2^ Institute of Molecular BiosciencesUniversity of GrazAustria; ^3^ Austrian Centre of Industrial BiotechnologyAustria

**Keywords:** electron transfer, flavin adenine dinucleotide, genetic disease, recombinant protein expression, X‐ray crystallography

## Abstract

The human dimethylglycine dehydrogenase (hDMGDH) is a flavin adenine dinucleotide (FAD)‐ and tetrahydrofolate (THF)‐dependent, mitochondrial matrix enzyme taking part in choline degradation, one‐carbon metabolism and electron transfer to the respiratory chain. The rare natural variant H109R causes dimethylglycine dehydrogenase deficiency leading to increased blood and urinary dimethylglycine concentrations. A detailed biochemical and structural characterization of hDMGDH was thus far hampered by insufficient heterologous expression of the protein. In the present study, we report the development of an intracellular, heterologous expression system in *Komagataella phaffii* (formerly known as *Pichia pastoris*) providing the opportunity to determine kinetic parameters, spectroscopic properties, thermostability, and the redox potential of hDMGDH. Moreover, we have successfully crystallized the wild‐type enzyme and determined the structure to 3.1‐Å resolution. The structure‐based analysis of our biochemical data provided new insights into the kinetic properties of the enzyme in particular with respect to oxygen reactivity. A comparative study with the H109R variant demonstrated that the variant suffers from decreased protein stability, cofactor saturation, and substrate affinity.

**Database:**

Structural data are available in the PDB database under the accession number 5L46.

**Abbreviations**
*Ag*DMGO: *Arthrobacter globiformis* dimethylglycine oxidase
DCPIP: 2,6‐dichlorophenolindophenol
DMG: dimethylglycine
DMGDH: dimethylglycine dehydrogenase
ETF‐QO: ETF‐ubiquinone oxidoreductase
hDMGDH: human dimethylglycine dehydrogenase
hETF: human electron‐transferring flavoprotein
hMCAD: human medium‐chain acyl‐CoA dehydrogenase
MRE: mean residue ellipticity
PMS: phenazine methosulfate
THF: tetrahydrofolate
WT: wild‐type

## Introduction

**Figure 1:**
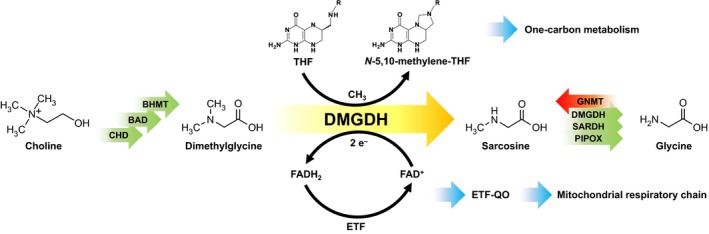
Mammalian choline degradation pathway with DMGDH as metabolic branch point. Choline degradation to dimethylglycine is catalyzed by three enzymes, choline dehydrogenase (CHD), betaine aldehyde dehydrogenase (BAD), and betaine homocysteine *S*‐methyltransferase (BHMT). Conversion of dimethylglycine (DMG) to sarcosine by DMGDH reduces the FAD cofactor and a methyl group is donated to THF resulting in FADH_2_ and *N*‐5,10‐methylene‐THF. The enzyme is reoxidized by the interaction with hETF and the derived electrons are further channeled by ETF‐QO to the mitochondrial respiratory chain. *N*‐5,10‐methylene‐THF is consumed in further reactions catalyzed by methylene‐THF reductase (MTHFR) and serine hydroxymethyltransferase (SHMT). DMGDH is the only enzyme converting dimethylglycine to sarcosine and the only other known route to sarcosine is via glycine *N*‐methyl transferase (GNMT). Sarcosine is further converted to glycine, mostly by sarcosine dehydrogenase (SARDH), and is also a substrate for dimethylglycine dehydrogenase and peroxisomal sarcosine oxidase (PIPOX).

The human flavoproteome comprises 90 enzymes with versatile functions, structures, and protein characteristics. Sixty percent of the known flavoproteins are involved in human diseases and disorders emphasizing their importance in human metabolism 1. The human mitochondrial matrix flavoprotein dimethylglycine dehydrogenase (hDMGDH, EC: 1.5.8.4) catalyzes the oxidative demethylation of dimethylglycine (DMG) to sarcosine, and also to a lesser extent the conversion of sarcosine to glycine as part of choline degradation (Fig. 1) 2, 3. Choline is an essential nutrient and building block in a variety of vital biomolecules such as the membrane phospholipid phosphatidylcholine and the neurotransmitter acetylcholine 4. Degradation of choline proceeds by consecutive oxidations via betaine aldehyde, betaine, dimethylglycine, and sarcosine to the amino acid glycine (Fig. 1) 5. hDMGDH, a key enzyme of this pathway, requires two cofactors: flavin adenine dinucleotide (FAD) and tetrahydrofolate (THF). The FAD is covalently attached via its 8α‐position to the N3 of a histidyl residue and serves as the electron acceptor in the oxidation of DMG. On the other hand, THF is used as the acceptor of the incipient methyl group and thus prevents the release of cell‐toxic formaldehyde during catalysis 3, 6. In the course of this reaction, *N*‐5,10‐methylene tetrahydrofolate is formed, which plays an important role in one‐carbon metabolism. Regeneration of oxidized FAD is achieved by electron transfer to the human electron‐transferring flavoprotein (hETF), which in turn transfers the electrons to the membrane‐anchored ETF‐ubiquinone oxidoreductase (ETF‐QO) for further utilization in the mitochondrial respiratory chain 7, 8.

A rare naturally occurring point mutation found in 58 out of 118 656 (0.049%) analyzed human gene sequences (Exome Aggregation Consortium online browser; http://exac.broadinstitute.org/variant/5-78351682-T-C) results in the exchange of histidine 109 to arginine (H109R). This variant reportedly suffers from lower stability and enzymatic activity 9, 10. The observed phenotype was described as dimethylglycine dehydrogenase deficiency with significantly higher levels of dimethylglycine in human body fluids causing muscle fatigue and a fish‐like odor (OMIM: 605850). Interestingly, the mutation is predominantly found in individuals of African descent.

In recent years, also the product of the reaction catalyzed by hDMGDH, sarcosine, attracted attention as a biomarker for aggressive prostate cancer 11, 12. Therefore, investigations on the regulation of sarcosine levels and the enzymes involved are in the focus of research. Furthermore, a recent epidemiological study revealed a possible connection of dimethylglycine dehydrogenase deficiency to the development of diabetes, further emphasizing the importance of the enzyme 13.

Dimethylglycine dehydrogenase (DMGDH) was first identified and purified from rat liver in the 1950s and early 1960s 14, 15. Since then several publications have dealt with the characterization of mammalian dimethylglycine dehydrogenases from rat 2, 3, 16 and pig liver 8 as well as the recombinant rat 17, 18 and human enzyme 19 using *Escherichia coli* as expression host. Furthermore, dimethylglycine oxidase from *Arthrobacter globiformis* (*Ag*DMGO) was characterized in biochemical and structural detail 6, 20, 21, 22, 23. However, the available information on the hDMGDH appears insufficient and partly contradictory. In order to overcome these deficiencies, we present a profound analysis of the recombinant hDMGDH and the H109R variant concerning kinetics, redox behavior, and spectral properties. Furthermore, we have elucidated the crystal structure of the human wild‐type enzyme.

In order to characterize hDMGDH, we have established a recombinant expression system for the wild‐type and the H109R variant in *Komagataella phaffii* (formerly known as *Pichia pastoris* or *Komagataella pastoris* 24) and a subsequent purification protocol. This approach enabled us to determine key kinetic parameters as well as physical and spectroscopic properties of wild‐type DMGDH. Consequently, we have also employed our expression system to generate the H109R variant to address the putative loss of function that leads to DMGDH‐deficiency in humans. Moreover, successful crystallization of the WT and subsequent crystallographic analysis provided us with the opportunity to analyze the structure with regard to substrate binding and oxygen reactivity. The latter issue is especially interesting as the control of oxygen reactivity in flavoenzymes is still a controversial topic. The direct comparison of a dehydrogenase (hDMGDH) and an oxidase (*Ag*DMGO) allowed new insights into the structural elements involved in oxygen reactivity and supports concepts that are based on gatekeeper residues in the vicinity of the isoalloxazine ring system. In this vein, we show that a previously proposed model to rationalize oxygen reactivity in the vanillyl oxidase family is applicable to the family of sarcosine and dimethylglycine dehydrogenases and oxidases, respectively 25, 26.

## Results

### Enzyme expression and purification

**Figure 2:**
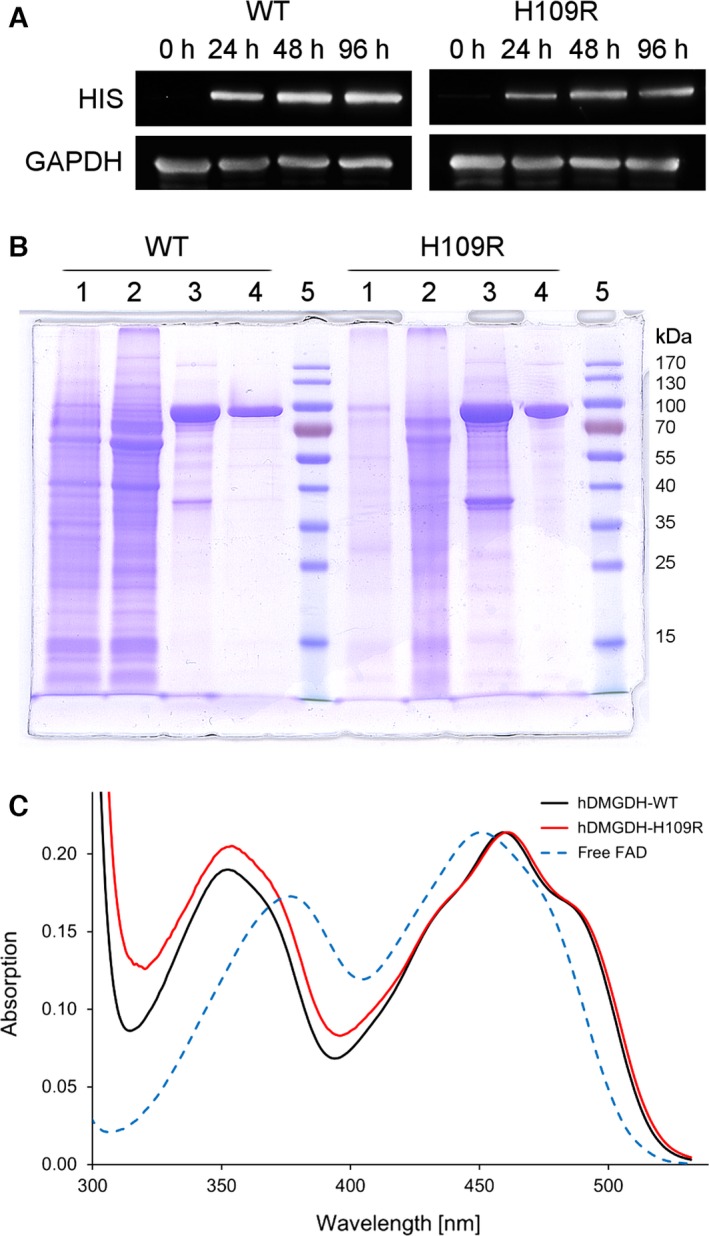
Heterologous expression and purification of hDMGDH‐WT and hDMGDH‐H109R variant. (A) Western blot of *K. phaffii* cell lysates taken at different time points after methanol induction. Antibodies are directed against a C‐terminal nona‐histidine tag fused to the recombinant enzymes and, as loading control, against *K. phaffii* GAPDH. (B) SDS/PAGE of the different purification steps of WT and variant shows efficient enzyme purification with Ni‐NTA affinity and MonoQ anion exchange chromatography. *Lane 1*, fermentation pellet fraction; *2*,* K. phaffii* lysate; *3*, enzyme fraction after Ni‐NTA affinity chromatography; *4*, enzyme fraction after Mono Q; *5*, protein standard. (C) UV‐Vis absorption spectra of DMGDH wild‐type (black line) and the H109R variant (red line) after anion exchange chromatography. The spectra were normalized to the long‐wavelength absorption maxima (445 and 450 nm, respectively). Free FAD in solution is shown as a dotted blue line.

Initial attempts to express the gene encoding hDMGDH in *Escherichia coli* (BL21 DE3) yielded largely insoluble protein. On the other hand, heterologous expression in the methanotrophic yeast *Komagataella phaffi* (formerly known as *Pichia pastoris* 24) was successful. As shown in Fig. 2A, western blot analysis of *K. phaffii* cell lysates at different time points indicates a stable expression of the protein after induction with methanol (MeOH). Typically, fermentations were stopped after 96 h after MeOH induction resulting in 1.6–1.9 kg of wet cell pellet. A comparison of signal intensities showed that the WT was expressed in higher amounts than the variant under identical fermentation conditions thus leading to a higher yield of WT (Fig. 2A). After cell disruption and Ni‐NTA affinity chromatography, the yield of proteins (Fig. 2B, lanes 3) was approximately 70 and 25 mg of the WT and H109R variant, respectively (40 or 15 μg enzyme per g wet cell weight). In order to achieve higher purity for crystallization trials, the proteins were further purified using anion exchange chromatography (Fig. 2B, lanes 4) resulting in lower protein yields of 30 and 2 mg (17 or 1 μg enzyme per g wet cell weight) of WT and H109R variant, respectively. The protein loss mainly occurred during the necessary buffer change to lower salt concentrations after Ni‐NTA affinity chromatography required for the subsequent anion exchange chromatography. Although we experienced that hDMGDH tends to precipitate at low salt concentrations, other chromatographic methods, like size‐exclusion chromatography or hydrophobic interaction chromatography, were explored but did not give a similar purity.

The *A*_280_/*A*_450_ ratios of the highly pure protein fractions were usually between 14–16 and 20–25 for the WT and variant protein, respectively. The UV‐Vis absorption spectra show a clear difference between enzyme‐bound FAD and free FAD (Fig. 2C), whereas hDMGDH‐WT and the variant featured similar spectral properties with identical absorption maxima. In comparison with free FAD in solution, the UV‐Vis absorption maxima of FAD bound to either WT or the H109R variant exhibit a hypsochromic shift from 370 to 350 nm and a bathochromic shift from 445 to 460 nm (Fig. 2C).

### Determination of kinetic parameters for hDMGDH‐WT and H109R variant

**Figure 3:**
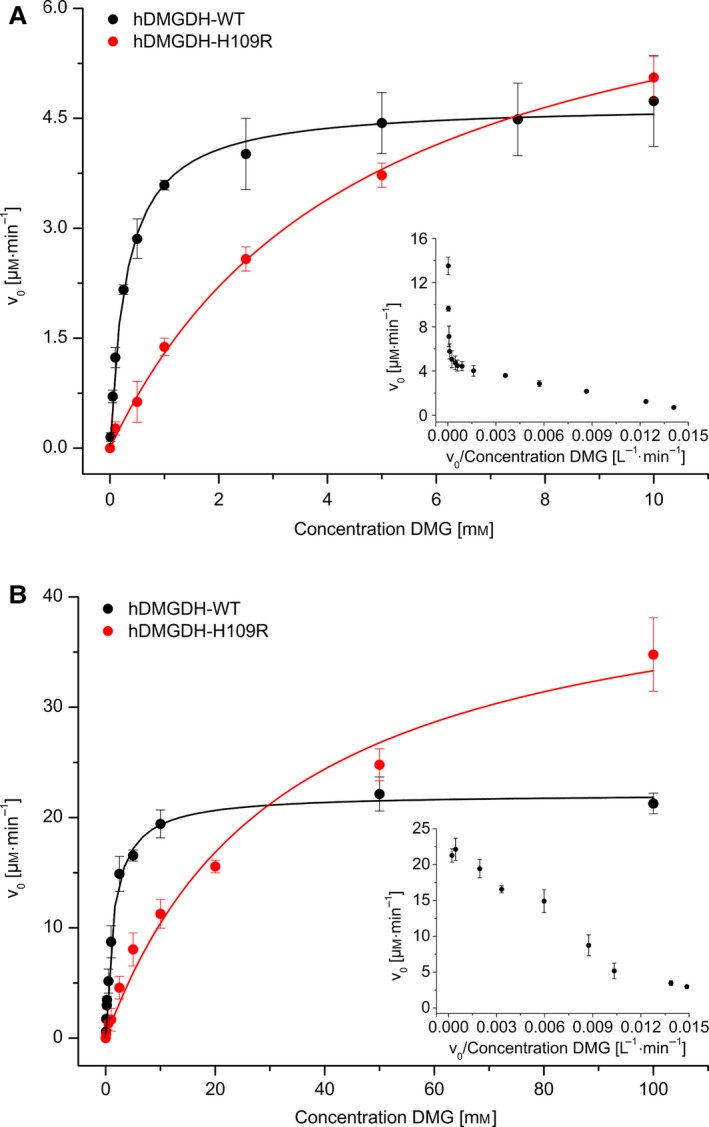
Steady‐state kinetics of hDMGDH‐WT and H109R variant. Steady‐state kinetic experiments were performed with DCPIP (A) and ferrocene activity assays (B) by plotting initial reaction velocities (*v*_0_) against the substrate concentration. Data obtained for WT and H109R variant are represented in black and red, respectively. Kinetic parameters are summarized in Table 1. Linearization of the data in Eadie–Hofstee diagrams reveals two *K*_M_ values for the DCPIP assay, one at low (0–10 mm DMG) and one at very high substrate concentrations (> 50 mm DMG, inset panel A). In contrast, the ferrocene assay produced a single *K*_M_ value when measured in the same concentration range (inset panel B). Error bars are shown as standard deviations; *n* = 3.

The evaluation of the steady‐state kinetic parameters for hDMGDH‐WT and H109R variant was conducted using either ferrocene or 2,6‐dichlorophenolindophenol (DCPIP) as electron acceptor to oxidize the reduced FAD cofactor. The obtained parameters show significant differences between the two assay systems both in terms of the *k*_cat_ as well as the *K*_M_ for DMG (Fig. 3, Table 1). When ferrocene was used as the artificial electron acceptor the *k*_cat_ was fivefold greater than with DCPIP (213 ± 4 versus 44 ± 1 min^−1^, Table 1) and the *K*_M_ was significantly higher (1.4 ± 0.1 versus 0.3 ± 0.002 mm, Table 1). Interestingly, the H109R variant exhibited a twofold higher activity than the WT in both assays. At the same time, the *K*_M_ of DMG was strongly increased by a factor of 23 and 16 in the ferrocene and DCPIP assay, respectively, resulting in an approximately 10‐fold lower catalytic efficiency of the H109R variant (Table 1). Notably, when DCPIP was used as electron acceptor, data linearization using the method of Eadie and Hofstee revealed a second, much higher *K*_M_ value of ≈ 30 mm for DMG (inset Fig. 3A). As the rate at which FAD is oxidized by ferrocene is closer to the rate of flavin reduction, it appears that ferrocene is superior to DCPIP as electron acceptor.

**Table 1:** Summary of kinetic parameters obtained with steady‐state kinetics for hDMGDH‐WT and hDMGDH‐H109R variant with two different activity assays (DCPIP and ferrocene)

	*K*_M_ (mm)	*v*_max_ (μm·min^−1^)	*k*_cat_ (min^−1^)	*A*_spec_ (μmol·mg^−1^·min^−1^)	*k*_cat_/*K*_M_ (mm ^−1^·min^−1^)
WT DCPIP	0.3 ± 0.002 ~ 30^a^	4.7 ± 0.1 ~ 11^a^	44 ± 1	0.5 ± 0.01	146 ± 12
WT ferrocene	1.4 ± 0.1	22.1 ± 0.3	213 ± 4	2.0 ± 0.04	137 ± 16
H109R DCPIP	4.7 ± 0.4 ~ 90^a^	7.4 ± 0.3 ~ 22^a^	91 ± 4	1 ± 0.04	20 ± 2
H109R ferrocene	32.2 ± 5.6	44.1 ± 3.2	544 ± 40	6 ± 0.4	11 ± 1

a Two *K*_M_ and *v*_max_ values were obtained with the DCPIP, the higher was roughly estimated from Eadie–Hofstee plots (Fig. 3).

**Figure 4:**
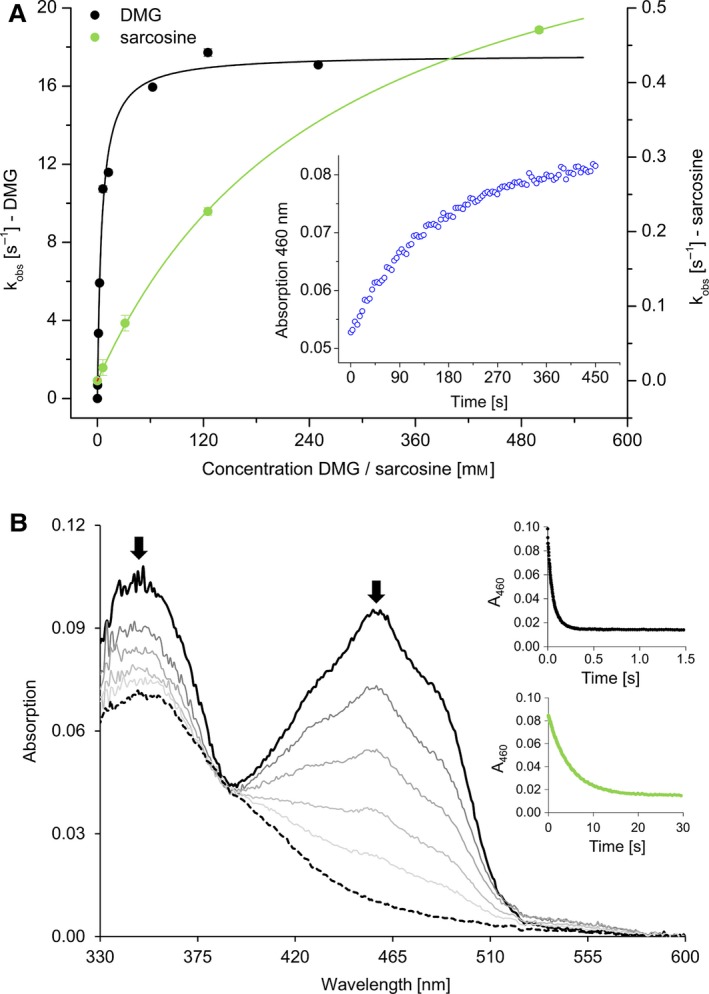
Presteady‐state kinetics of hDMGDH‐WT. Reductive and oxidative rates were measured under anoxic conditions with the stopped flow device. (A) Reductive rates were determined for DMG (black, left axis) and sarcosine (red, right axis). The inset shows the reoxidation of reduced FAD as a function of time at an oxygen concentration of 10.5% (135 μm O_2_) at 460 nm. At least three independent measurements were performed; error bars are shown as standard deviations. (B) Selected absorption spectra of the anoxic reduction of 10 μm hDMGDH‐WT with 125 mm DMG. The direction of the change in absorption is depicted by arrows. The top inset shows the absorption change at 460 nm observed by mixing WT with 125 mm DMG, and the bottom inset shows the change at 460 nm mixing WT with 125 mm sarcosine.

Due to much lower enzyme amounts available of the H109R variant, presteady‐state kinetics were solely measured with the wild‐type enzyme. As shown in Fig. 4A, the rate of reduction as a function of DMG concentration fitted to a hyperbolic equation yielding a limiting reductive rate of 17 ± 0.3 s^−1^ and a dissociation constant of 4.9 ± 0.3 mm for DMG. Thus, the reductive rate is 5–20 times faster than *k*_cat_ determined in the ferrocene and DCPIP (Table 1) indicating that the electron transfer from the reduced FAD to the artificial electron acceptor is rate‐limiting in the assays used to evaluate steady‐state kinetics. Reoxidation of hDMGDH with air‐saturated buffer proceeds very slowly with an observed rate of reoxidation of 0.006 ± 0.001 s^−1^ at an oxygen concentration of 135 μm (inset Fig. 4A). Rapid reaction measurements with sarcosine yielded a limiting rate of reduction of 0.7 ± 0.1 s^−1^ and a dissociation constant of 280 ± 10 mm clearly indicating that sarcosine is a much poorer substrate than DMG (Fig. 4A).

**Figure 5:**
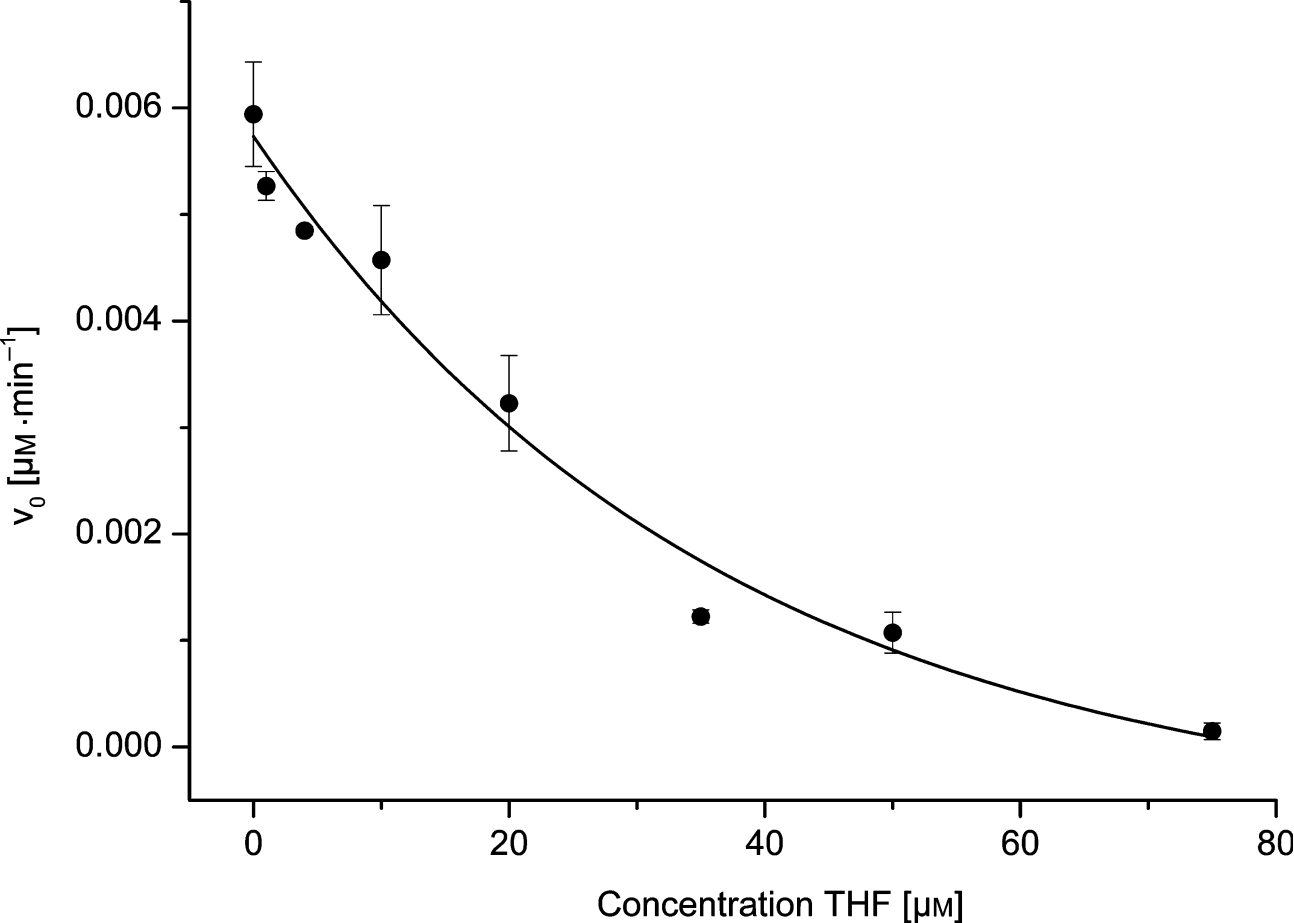
hDMGDH‐WT activity as a function of the THF concentration. The activity was measured using the ferrocene activity assay. The error bars show the standard deviation of at least three measurements for each THF concentration.

Finally, we also studied the effect of THF on the activity of hDMGDH using the ferrocene assay (Fig. 5). The activity measurements were done similarly to the ferrocene activity assay as described above at 25 °C and pH 7.8 in 50 mm HEPES/NaOH with 150 mm NaCl, 100 nm hDMGDH, 1 mm DMG, 0.1 mm EDTA, and 0.2 mm freshly prepared ferrocene in a concentration range of 0–75 μm THF. As shown in Fig. 5, the activity of WT decreases as a function of the THF concentration.

### Photoreduction and determination of the redox potential of hDMGDH

**Figure 6:**
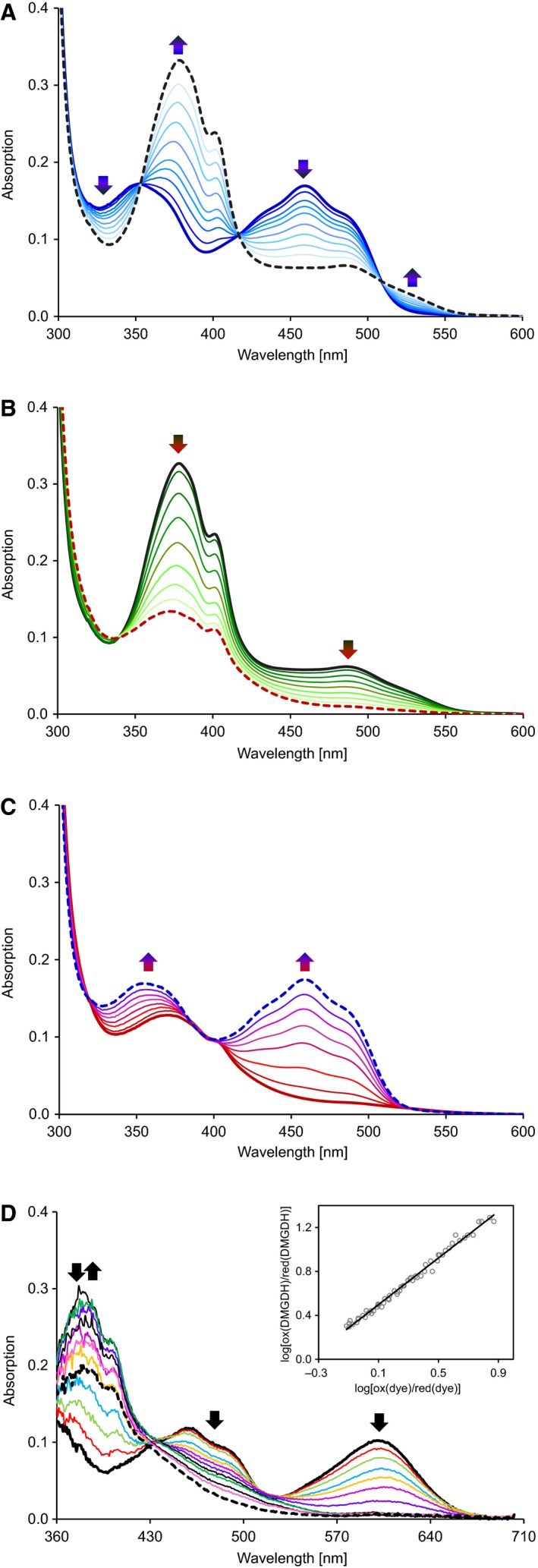
Photoreduction and redoxpotential of hDMGDH‐WT. (A) and (B) show selected absorption spectra of the anoxic photoreduction of the wild‐type enzyme. About 20 μm of hDMGDH was photoreduced in the presence of 1 mm EDTA, 2 μm 5‐deaza FMN, and 2 μm methylviologen. First, the enzyme is reduced to the red semiquinone radical state (A) before proceeding to the typical spectrum of a fully reduced hydroquinone species (B). The admission of oxygen to the enzyme shows that reoxidation does not proceed through a semiquinone redox state and directly yields the oxidized state (C). (D) The redox potential for the WT was determined with the xanthine/xanthine oxidase system described by Massey 27. The plot shows the simultaneous reduction of the enzyme and the dye with the double logarithmic evaluation of the data according to Minnaert 39 in the inset. About 20 μm hDMGDH were reduced at 25 °C over 120 min with indigotrisulfonic acid potassium salt. The evaluation for the enzyme was done at 480 nm where the dye does not show a contribution to the absorption. Values for the dye were measured at 600 nm. Initial absorption spectra in all panels are depicted in bold lines, whereas final absorption spectra are shown as dashed lines. Only selected absorption spectra are shown. The direction of the change in absorption is depicted by arrows.

Reduction of hDMGDH by DMG shows a monophasic conversion of the oxidized FAD to the two‐electron reduced dihydroquinone state (Fig. 4B) without any transient appearance of a semiquinone radical. In contrast to substrate reduction, photoreduction of hDMGDH leads to the anionic (red) flavin semiquinone with a typical absorption maximum at 372 nm (Fig. 6A). Further photoreduction eventually leads to the fully reduced dihydroquinone (Fig. 6B), which is directly converted to the oxidized state without the occurrence of a semiquinone upon reaction with molecular dioxygen (Fig. 6C). This was also seen when reduced hDMGDH was reacted with air‐saturated buffer in the stopped flow device (data not shown).

In order to determine the redox potential of hDMGDH, we employed the xanthine oxidase/xanthine method described by Massey 27 using indigotrisulfonic acid potassium salt as reference dye. The slope of the double logarithmic plot was close to unity indicating that the dye and the flavin receive an equal number of electrons (i.e. two), although the anionic semiquinone radical is clearly observable during the redox titration (Fig. 6D). The redox potential for the reduction of FAD bound to hDMGDH was calculated from six independent measurements to −93 ± 1 mV.

### Thermal stability of hDMGDH‐WT and hDMGDH‐H109R

**Figure 7:**
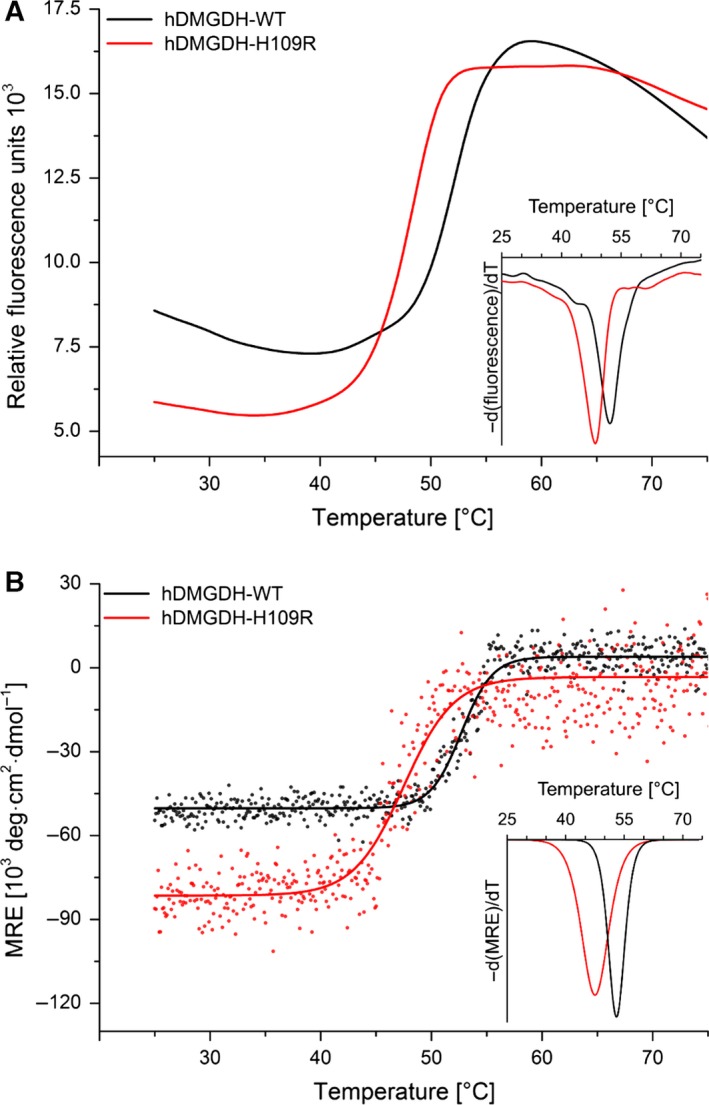
Thermal stability of hDMGDH‐WT and the H109R variant. The melting temperatures of wild‐type and the variant enzyme differ significantly when measured with Thermofluor^®^ (A) or CD spectroscopy (B). Thermofluor^®^ was done with 2 μm enzyme and the addition of SYPRO Orange. The change in fluorescence during heating is shown for WT and H109R variant in black and red lines, respectively. The inset in panel A shows the −d(fluorescence)/dT derivative curves of the raw data. CD spectroscopy was done with approximately 6 μm enzyme. Mean residue ellipticity (MRE) was measured every 0.1 °C at 208 nm which is shown by black‐ and red‐filled circles for WT and variant, respectively. The data were fitted with a sigmoidal curve in origin. The inset in panel B shows the −d(MRE)/dT derivative curves of the sigmoidal fits. The variant (red line) exhibits an inflection point at a lower temperature as the wild‐type (black line) in both measurements.

The thermal stability of both enzymes was determined using Thermofluor^®^ and circular dichroism spectroscopy. Thermofluor^®^ temperature scans from 25 to 95 °C showed that the WT and the H109R variant have melting points (*T*_m_) of 52 °C and 47 °C, respectively (Fig. 7A). A similar result was obtained by means of CD spectroscopy yielding a *T*_m_ of 53 °C and 47 °C, for WT and the variant, respectively (Fig. 7B). Thus, both independent methods confirm that the variant exhibits reduced thermal stability.

### Protein structure analysis

**Figure 8:**
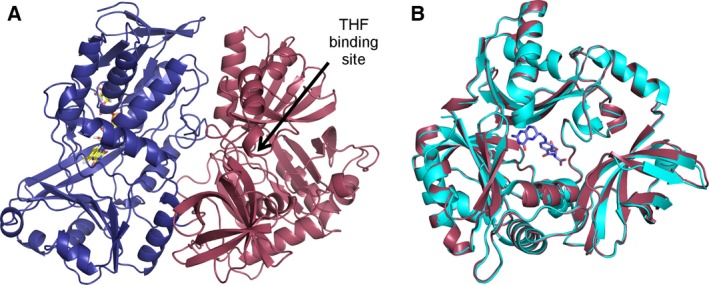
Crystal structure of hDMGDH. (A) The overall structure of hDMGDH with the FAD‐binding domain is shown in deep blue and the folate‐binding domain in raspberry. The THF‐binding site is depicted with an arrow and the FAD cofactor is shown in yellow sticks. (B) Superposition of the folate‐binding domains of DMGDH from human (raspberry) and rat (cyan). The THF cofactor is shown in blue sticks representation.

The X‐ray crystal structure of hDMGDH was determined at 3.1 Å resolution (Table 2). The crystal belongs to the monoclinic space group (*P*2_1_) and contains two dehydrogenase molecules in the asymmetric unit. The two molecules in the asymmetric unit are very similar to each other with an rmsd of 0.22 Å calculated after the superposition of 810 out of 828 Cα‐atoms using the program pymol (DeLano Scientific). As previously observed in rDMGDH and also after superposition with the rat enzyme (rmsd of 0.37 Å calculated for 739 out of 809 Cα‐atoms), the human enzyme consists of two domains: the FAD‐binding domain (N‐terminal domain with residues 46–466) and the folate‐binding domain (C‐terminal domain with residues 467–855, Fig. 8A). The FAD cofactor is covalently attached to the protein through a linkage between N3 of His91 and the 8α‐methyl group of the isoalloxazine moiety as confirmed by the crystal structure. The folate‐binding domain can be divided into three subdomains positioned in a ‘cloverleaf’‐like arrangement as previously described for the rat enzyme 18. Subdomain 1 (residues 467–537 and 626–728) contains a Greek‐key motif surrounded by α‐helices. Subdomain 2 (residues 538–625 and 729–770) is defined by a five‐stranded antiparallel β‐sheet with flanking α‐helices. Subdomain 3 (residues 771–850) adopts a jellyroll fold. The structure of the rDMGDH was determined in complex with THF 18. We also observed some difference electron density in the same area in the folate‐binding domain. Due to the lower resolution of the diffraction data and/or an incomplete occupancy of the ligand, however, this density was not clear enough to unequivocally position a folate molecule. Based on the structural similarity of the two enzymes, it is still very likely that folate binds in the same position (Fig. 8B).

**Table 2:** Data collection and refinement statistics

X‐ray source	ID29, ESRF, Grenoble, France
Wavelength (Å)	0.972
Temperature (K)	100
Space group	*P*2_1_
Cell dimensions	
*a*,* b*,* c* (Å)	83.38, 119.87, 86.47
β (°)	92.59
Resolution (Å)	
High resolution shell	58.65–3.09 (3.2–3.09)
Total no. reflections	86 727 (7935)
Unique no. reflections	30 495 (2897)
Multiplicity	2.8 (2.7)
Completeness (%)	97.7 (93.81)
<I/σI>	4.81 (1.53)
Wilson B‐factor	47.09
*R*_merge_	0.2058 (0.6573)
*R*_meas_	0.2531
CC_1/2_	0.958 (0.604)
CC*	0.989 (0.868)
*R*_work_/*R*_free_	17.90/26.93
Number of nonhydrogen atoms	
Macromolecules	12 782
Ligands	106
Water	6
Protein residues	1617
Average B‐factor	38.1
Macromolecules	38.2
Ligands	30.1
Solvent	15.2
R.m.s. deviations	
Bond lengths (Å)	0.010
Bond angles (°)	1.37
Ramachandran outliers (%)	0.31
Ramachandran favored (%)	94
Clashscore	9.40
PDB	5L46

## Discussion

### Heterologous enzyme expression and purification

In this study, we demonstrated that intracellular expression of hDMGDH in *K. phaffii* is feasible and provides enough hDMGDH to perform a detailed biochemical and structural characterization. The expression of the full‐length protein and a Δ50 truncated version as well as secretory expression were unsuccessful. However, an N‐terminal Δ28 truncation of the enzyme was expressed successfully representing the mature form of the enzyme lacking its mitochondrial targeting sequence 28. The intracellular coexpression of the *Saccharomyces cerevisiae* protein disulfide isomerase enzyme resulted in improved protein yields. The expression of hDMGDH in *E. coli* as reported before 19 was unsuccessful and failed to yield detectable amounts of protein as judged by western blotting. A C‐terminal nona‐histidine‐tag was added to the gene in order to facilitate purification by means of affinity chromatography. According to the previously published structure of the rat enzyme 18, this C‐terminal tag should not interfere with the native fold of the protein or catalytic activity. Western blot analysis showed that the H109R variant is expressed in lower amounts compared to the WT (Fig. 2A).

A theoretical *A*_280_/*A*_450_ ratio for hDMGDH fully saturated with FAD cofactor can be roughly calculated to 14. The observed *A*_280_/*A*_450_ ratios of the highly pure proteins, 14–16 and 20–24 for WT and H109R variant, respectively, revealed that the WT is almost fully loaded with the covalently attached FAD. In the case of the variant, the ratio was much higher indicating a much lower saturation compared to the WT (ca. 40–50% apoprotein), similar to previously reported observations 19. The higher fraction of apoprotein may be responsible for the recurring protein precipitation during purification of the variant. Overall, this led to substantially reduced yields of the variant protein and limited the scope of experiments that could be conducted.

### Spectral characteristics and steady‐state kinetics of hDMGDH

**Figure 9:**
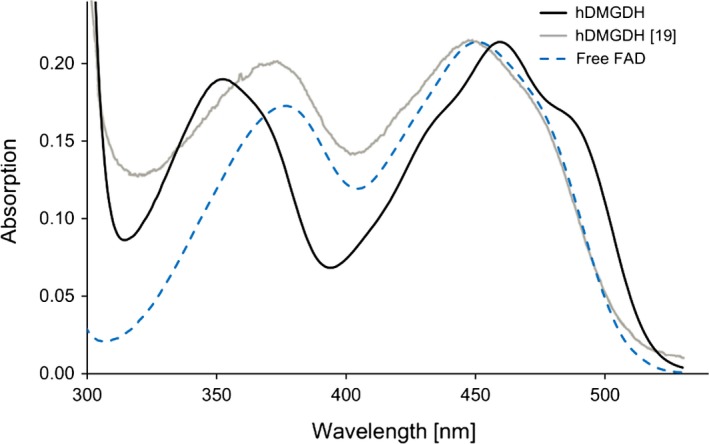
Protein spectrum of this study (black line) compared with the published hDMGDH spectrum (gray line, 19) and free FAD in solution (dotted blue line)**.** The published spectrum resembles free FAD in solution and not our spectral findings.

Recombinant hDMGDH features characteristic UV‐Vis absorption properties that clearly distinguish the bound FAD from FAD free in solution (Fig. 2C). These spectral characteristics are very similar to those reported earlier for DMGDH isolated from rat and pig liver as well as for recombinant *Ag*DMGO 8, 16, 20. In contrast, the UV‐Vis absorption spectra for heterologously expressed rat DMGDH 17 and human DMGDH 19 are either of very poor quality or apparently lack the spectral features that are typically found in these enzymes. In fact, the only published spectrum of hDMGDH expressed in *E. coli* is very similar to free FAD in solution (compare spectra shown in Fig. 9).

A summary of our data and the previously published kinetic steady‐state data is given in Table 3. These values were obtained using two different enzymatic activity assays, on one hand with ferrocenium hexafluorophosphate as electron acceptor and on the other hand, the combination of phenazine methosulfate (PMS) as electron mediator and DCPIP as terminal electron acceptor. Both assays are physiologically irrelevant as all mammalian DMGDHs interact with the electron‐transferring flavoprotein (hETF) for reoxidation of the reduced cofactor. With the ferrocene assay, we have obtained comparable results as reported for the rat enzyme 18 and also for the bacterial DMGO 22. However, analysis of the data obtained with the PMS/DCPIP yielded two Michaelis–Menten constants similar to previously published data for the rat enzyme 16, 17. Interestingly, aside from the results reported by McAndrew *et al*. 19, two *K*_M_ values were only observed for the two component DCPIP activity assay, with PMS as electron mediator, but not in the one component ferrocene or H_2_O_2_ assay suggesting that it is an experimental artifact. In view of this apparent inconsistency, speculations that the presence of two *K*_M_ values reflect the presence of activating or regulatory sites in the enzyme are unwarranted 16, 17. Moreover, we have shown that the rate of enzyme reduction is faster than *k*_cat_ indicating that reoxidation is rate‐limiting for turnover in both assays. Thus, the affinity of DMG to hDMGDH is more reliably expressed as the dissociation constant determined in presteady‐state experiments.

**Table 3:** Comparison of steady‐state kinetic parameters of published results for the DMGDH from *Homo sapiens* (human), *Rattus norvegicus* (rat), *Sus scrofa* (pig), as well as the dimethylglycine oxidase from *A. globiformis* (*Ag*)

Enzyme	e^‐^‐Acceptor	Conditions	*K*_M_ (mm)	*k*_cat_ (min^−1^)	*A*_spec_ (μmol·mg^−1^·min^−1^)	*k*_cat_/*K*_M_ (mm ^−1^·min^−1^)
Human – this study	DCPIP	pH 7.8, 25 °C	0.3 ± 0.002^b^	44 ± 1	0.5 ± 0.01	146 ± 12
Human – this study	Ferrocene	pH 7.8, 25 °C	1.4 ± 0.1	213 ± 4	2.0 ± 0.04	137 ± 16
Human – 19	Ferrocene	pH 7.5, 25 °C	0.04 ± 0.01^b^	18 ± 2	0.165^a^	450 ± 110
Rat – 18, a	Ferrocene	pH 7.5, 25 °C	0.6	160	nd	270
Rat – 17, a	DCPIP	pH 7.0, 30 °C	0.05^b^	12.1	0.24	nd
Rat – 16, a	DCPIP	pH 7.0, 25 °C	0.05^b^	8.4	0.14	nd
Pig – 8, a	DCPIP	pH 7.0, 25 °C	nd	nd	0.157	nd
*Ag* – 22	O_2_	pH 8.5, 25 °C	2.4 ± 0.2	640 ± 20	nd	270 ± 32

nd, Not determined or stated in the publication. ^a^ No standard deviations available. ^b^ Two *K*_M_ values were observed but only the physiologically relevant is displayed.

### Effects of a naturally occurring H109R enzyme variation

The rare naturally occurring enzyme variant H109R causes a nonfatal disease called dimethylglycine dehydrogenase deficiency (OMIM: 605850). The defect results in an accumulation of DMG in blood serum and urine, and patients suffer from a mild phenotype including muscle fatigue and fish‐like odor 9, 10. During heterologous expression in *K. phaffii*, we noticed decreased expression levels resulting in much lower yields of the variant protein. Further characterization of the variant protein demonstrated lower cofactor saturation and thermal stability. Interestingly, the variant showed elevated turnover rates in both of our assay systems although *K*_M_ values were 15–25 times higher and the catalytic efficiency 10‐fold lower than for the WT (Table 1). Hence, our results are clearly in contradiction with previously published data 19 that claimed significantly decreased catalytic activity of the H109R variant. As the spectral properties of DMGDH preparations obtained from the *E. coli* expression system lacks the characteristic features typically observed for DMGDH isolated from natural sources (e.g., rat and pig liver), it is conceivable that recombinant DMGDH generated in bacterial host cells suffers from an altered FAD‐binding mode, such as noncovalent binding. In fact, the absorption spectrum reported for wild‐type DMGDH and its H109R variant is clearly more similar to that of free FAD (Fig. 9). In our hands, the bacterial expression system produced solely insoluble protein and therefore analysis of FAD binding to the protein could not be inspected further.

**Figure 10:**
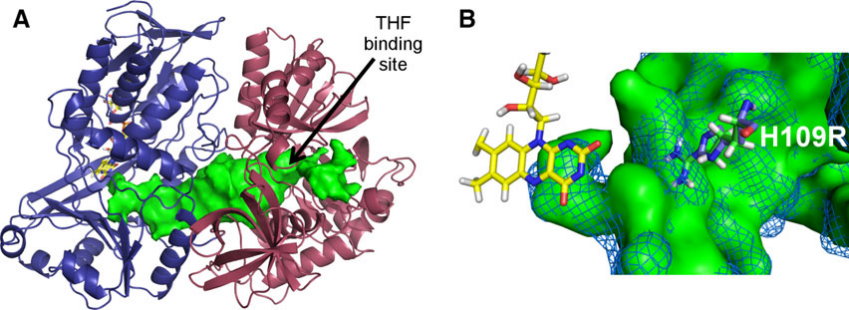
hDMGDH internal cavity analysis and effect of H109R variation. (A) Cavity analysis of hDMGDH done with CASoX in pymol without a bound THF cofactor. The protein cavity is shown in green surface view and the THF‐binding site indicated by an arrow. (B) Close‐up view of the site of amino acid replacement (i.e., H109R) near the active site of the protein. A cavity analysis at this site was done in the wild‐type enzyme (His‐109, blue mesh) and the H109R variant (Arg‐109, green surface). The bulky, charged arginine extends into the cavity calculated for the wild‐type protein and potentially reduces the size of the substrate tunnel, thus substrate delivery to the FAD‐binding site. FAD cofactor is shown in yellow, the residues at position 109 in green and blue sticks, respectively.

Previous reports on the structure of bacterial DMGO and rat DMGDH identified a large internal cavity in the protein 6, 18. This cavity serves as ‘reaction chamber’ responsible for substrate delivery to the active site, substrate conversion, as well as intermediate channeling to THF and product formation. The binding site of THF is the only entry‐exit point of the protein, which was revealed by MD simulations for the structurally related DMGO 6 and also confirmed by cavity analysis of our structure (Fig. 10A). In accordance with this interpretation, increasing THF concentrations exhibited a negative effect on the turnover rate (Fig. 5) as the substrate DMG competes with THF for entrance into the cavity at the THF‐binding site.

The consistently (16‐ to 23‐fold) higher *K*_M_ observed in both steady‐state assays with the variant suggests that replacement of histidine by the bulkier arginine impedes access of DMG to the binding site (Fig. 10B). On the other hand, the H109R variant possessed higher *k*_cat_ values indicating that the variant is more permissible in transferring electrons from the reduced FAD to the artificial electron acceptors.

In conclusion, our analysis of the disease‐related H109R variant has revealed a lower thermal stability and an approximately 10‐fold lower catalytic efficiency (Table 1) as major effects of the single amino acid exchange. The diminished protein expression and cofactor saturation found in our expression system may also be relevant for homologous expression of the variant in human cells and thus contribute to the overall reduced DMGDH activity manifested in affected human subjects.

### Substrate specificity, redox behavior, and active site composition

**Figure 11:**
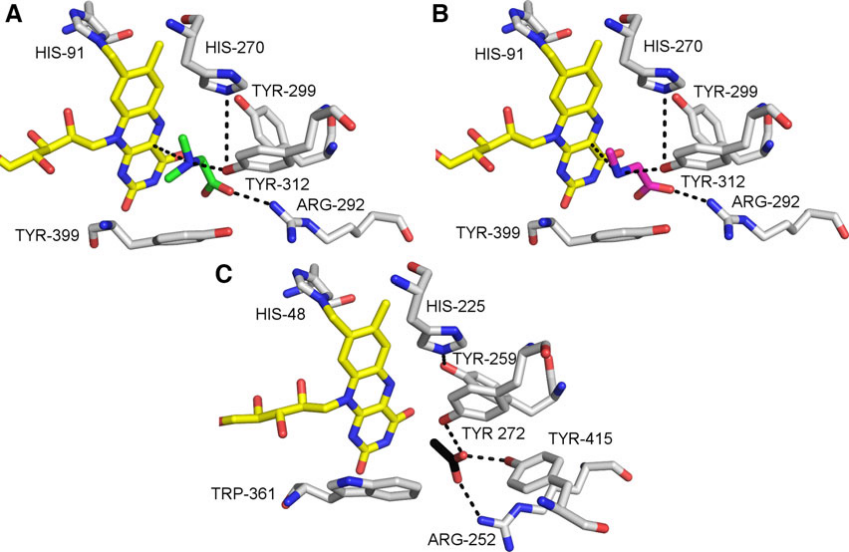
Docking and active site composition of hDMGDH in comparison with bound acetate in DMGO structure. DMG (A) and sarcosine (B) were docked into the active site of hDMGDH with autodock/vina 47. The active site of DMGO with bound acetate (C) was retrieved from PDB ID: 1PJ5. The docked substrates and the bound acetate are shown in green, magenta, and black. FAD is shown in yellow and all residues are depicted in stick representation colored according to the atom type. Key hydrogen bonds are shown with black dotted lines.

The overall protein structure and the active site of hDMGDH with the covalently bound FAD as well as the THF‐binding site are very similar to the rat enzyme (PDB ID: 4PAA; 18), and the structure of *Ag*DMGO 21. In 2003, *Ag*DMGO could be crystallized with a bound folic acid (PDB ID: 1PJ6) but additionally also with a bound acetate ion that mimics the negatively charged carboxylate group of DMG (PDB ID: 1PJ5). Most residues in the active site of *Ag*DMGO described to play a role in catalysis and substrate binding are also present in hDMGDH (Fig. 11). Docking of DMG and sarcosine to the active site of the hDMGDH structure results in a similar positioning of both substrates (Fig. 11A,B) and thus do not rationalize the differences observed in affinity and catalytic activity. This binding mode is similar to the acetate moiety in the crystal structure of DMGO (Fig. 11C). Presteady‐state measurements comparing DMG and sarcosine as substrate for hDMGDH‐WT show a 25‐fold decrease of the reductive rate for sarcosine as the substrate and a more than 50‐fold higher *K*_D_. These results are in very good agreement with data obtained for the bacterial DMGO (Table 4) 22. Therefore, sarcosine is a poor but possibly physiologically relevant substrate for the enzyme as already shown previously for the pig enzyme 3 and *Ag*DMGO 22. On the other hand, sarcosine is apparently oxidized by a homologous sarcosine dehydrogenase (SARDH) 16 and also by peroxisomal sarcosine oxidase (PIPOX) 29. Therefore, the relative turnover of sarcosine by these three FAD‐dependent enzymes must await further studies.

**Table 4:** Summary and comparison of transient kinetic data of hDMGDH (this study) with published results from *A. globiformis* DMGO 22, 23. Measurements were done in a stopped flow at pH 7.8 and 25 °C for hDMGDH and 25 °C and pH 8.5 for the bacterial enzyme

	*K*_D,DMG_ (mm)	*k*_red,DMG_ (s^−1^)	*K*_D,SAR_ (mm)	*k*_red,SAR_ (s^−1^)	*k*_ox_ (mm ^−1^·s^−1^)	*k*_obs,ox_ (s^−1^)
hDMGDH	4.9 ± 0.3	17 ± 0.3	280 ± 10	0.7 ± 0.1	0.04 ± 0.01	0.006 ± 0.001^b^
*Ag*DMGO	~ 10^a^	15 ± 0.2	850 ± 100	0.16 ± 0.1	201	26^c^

^a^ Retrieved from Table 2 in 23. ^b^ Measured at an oxygen concentration of 135 μm with p_O2_ = 0.105 bar, K_H,pc_ = 791 bar m ^−1^ 49 for the substrate reduced enzyme. ^c^ Estimation from fig. 8 in 22.

In the bacterial enzyme, a catalytic dyad, consisting of a histidine and a tyrosine, is responsible for deprotonation of the substrate amine 21. This catalytic dyad is also present in the human enzyme (Fig. 11). Furthermore, residues interacting with DMG (and sarcosine) were identified in the structure of hDMGDH (Fig. 11A). After binding and conversion of the substrate at the active site, an intermediate, most likely a cyclic lactone is channeled through the protein cavity to the THF‐binding site 6. The THF‐binding sites of mammalian and bacterial enzymes are again very similar and were already extensively discussed by Luka *et al*. 18.

Basran *et al*. reported a multistep kinetic model for the reduction of DMGO by DMG. In contrast, the reduction of hDMGDH by DMG was adequately fit by a single exponential equation. The actual measurement of the intermediate cyclic lactone could not be observed, most likely due to its short lifetime as described elsewhere 6.

The two dehydrogenases in choline degradation belong to a family of enzymes that delivers electrons to the electron transferring flavoprotein (ETF). Among these clients, only the redox potential for the two‐electron reduction of human medium‐chain acyl‐CoA dehydrogenases (hMCAD) was previously determined 30, 31. This prompted us to determine the redox potential for the covalently bound FAD in hDMGDH. The obtained value of −93 ± 1 mV is 40 mV more positive than that of hMCAD (−135 mV 30, 31) in agreement with the fact that covalent linkages to the 8‐methyl group increase the redox potential 32. In any case, the much higher redox potential of hETF (+37 mV for the oxidized/semiquinone couple, 33) ensures that electrons from hDMGDH are readily transferred to the ETF‐bound FAD.

The rates of reduction determined for hDMGDH are in good agreement with those observed for bacterial DMGO (Table 4). In contrast to that, the oxygen reactivity of reduced hDMGDH is more than 300 times lower and apparently physiologically irrelevant as reoxidation by its cognate partner protein hETF proceeds over 30 times faster (P. Augustin, E. Gerstmann and P. Macheroux, unpublished results).

**Figure 12:**
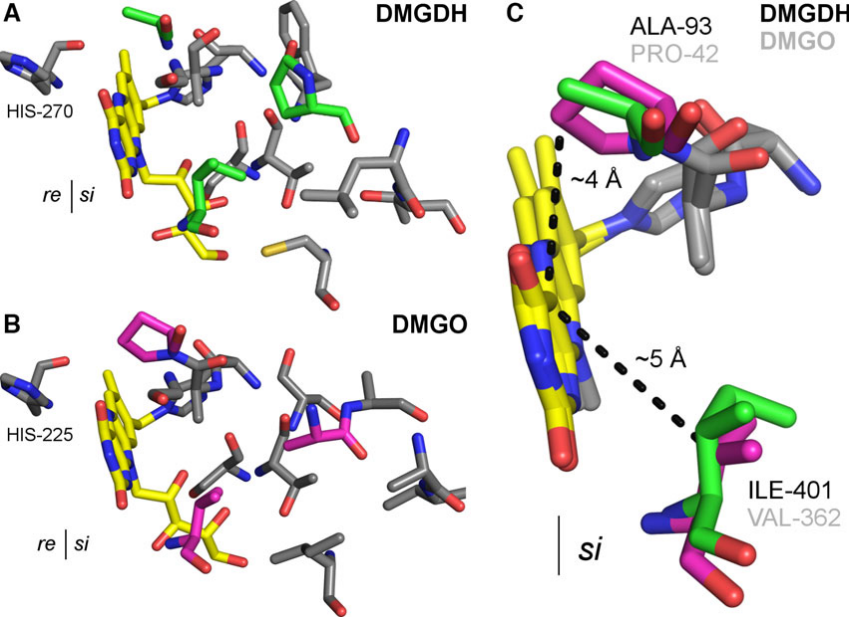
Active site composition of the oxygen‐binding site of hDMGDH and *Ag* DMGO. The human structure is shown in panel A with the most significant alternated residues on the *si‐*face of the flavin isoalloxazine ring shown in green sticks. The bacterial structure is shown in panel B, important residues are shown in magenta. For a better orientation, the catalytic active histidine on the *re‐*face is also displayed. Panel C shows the alignment of the bacterial and the human enzyme with the supposed gatekeeper residues shown corresponding to the reactive C(4a) carbon of the isoalloxazine ring. All residues are depicted in stick representation, FAD in yellow and colored according to the atom type.

Recently, a study concerning steric control of dioxygen reduction in enzymes of the vanillyl‐alcohol oxidase (VAO) family discovered a new ‘gatekeeper’ residue in berberine bridge enzyme and the pollen allergen Phl p 4 25. This gatekeeper appears to control access to an oxyanion hole essential to stabilize reaction intermediates during oxidation of the reduced isoalloxazine ring by dioxygen. In this family, valine was found to grant access to the oxyanion hole, whereas isoleucine denies access 25. Similarly, Leferink *et al*. discovered that a large number of oxidases in the VAO family contain either a glycine or a proline at a structurally conserved position near the isoalloxazine ring 26. Although hDMGDH does not belong to the VAO protein superfamily, comparison of the hDMGDH to the *Ag*DMGO structure reveals that hDMGDH features residues typical for dehydrogenases suppressing oxygen reactivity, i.e., an alanine and isoleucine, whereas *Ag*DMGO possesses residues compatible with high oxygen reactivity, i.e., proline and valine (Fig. 12). Nevertheless, in order to further support, if the emerging concept of how oxygen reactivity is controlled in flavoproteins is applicable to DMGDH, a member of the d‐amino acid oxidase (DAO) family of FAD‐dependent oxidoreductases (pfam: 01266), a detailed mutagenesis study of the concerned residues has to be conducted.

## Experimental procedures

### Enzymes and reagents

Restriction enzymes and Phusion DNA polymerase were from Thermo Fisher Scientific (Waltham, MA, USA), purification columns and materials from GE Healthcare (Chalfont St Giles, UK) and salt‐free purified oligos for site‐directed mutagenesis from VBC‐Biotech (Vienna, Austria). Antibodies used for western blot were from Cell Signaling Technology (Cambridge, UK). The redox dye for the determination of the redox potential, indigotrisulfonic acid potassium salt, was from TCI Europe N.V. (Zwijndrecht, Belgium). All other chemicals and media were from Carl Roth GmbH (Karlsruhe, Germany) and Sigma‐Aldrich (St. Louis, MO, USA) and were of the highest grade available.

### hDMGDH expression strain generation

hDMGDH and hDMGDH‐H109R expression plasmids were designed for intracellular enzyme expression in *Komagataella phaffii* KM71H strain (formerly known as *Pichia pastoris* or *Komagataella pastoris* 24). The hDMGDH sequence was codon optimized for expression in *K. phaffii* using GeneOptimizer^®^ (Thermo Fisher Scientific). In accordance to Binzak *et al*., the sequence of the mature form of hDMGDH (lacking the first 28 amino acids, 28) was used, flanked with *Xho*I and *Not*I restriction sites. For enzyme expression and purification, a Kozak sequence in front of the start codon (AAAA) and a C‐terminal nona‐histidine tag were added. The designed gene was cloned into a pPICZ B expression vector (Thermo Fisher Scientific) and verified by automated sequencing. The recombinant plasmid was transformed to *K. phaffii* KM71H strain (Thermo Fisher Scientific), harboring a pPICK‐PDI vector containing the gene for the protein disulfide isomerase from *Saccharomyces cerevisiae* following the guidelines of the Invitrogen EasySelect^™^ *Pichia* Expression Kit (Thermo Fisher Scientific). Positive clones were selected by Zeocin resistance and verified by western blot. The H109R variant was constructed by a two‐step site‐directed mutagenesis method employing Phusion DNA polymerase and 5′‐AACTTGAAGAAGATCAGATACGACTCCATCAAG‐3′ as forward and 5′‐CTTGATGGAGTCGTATCTGATCTTCTTCAAGTT‐3′ as reverse primer (alternated codon underlined). For the first reaction step, two separate PCR reactions using either the forward or the reverse primer were run with the PCR program: 98 °C (2 min) – [98 °C (50 s) – 60 °C (20 s) – 68 °C (16.5 min)] × 5 – 4 °C ∞. Afterwards, the two PCR reactions were combined and the same program was employed for another 20 cycles. Then, after a 2‐h *Dpn*I digestion step, the plasmid was transformed to *Escherichia coli* TOP10 cells for amplification before transformation to *K. phaffii* KM71H [pPICK‐PDI]. Strain selection was done as described above.

### Enzyme expression

hDMGDH and hDMGDH‐H109R expression was carried out in a 7‐L glass fermenter BBI CT5‐2 system (Sartorius, Göttingen, Germany). Preparation of inoculum, and preparation of the fermenter, batch, and fed‐batch were carried out as described by Schrittwieser *et al*. 34. The fermentation medium was a minimal basal salt medium (MgSO_4_·7 H_2_O – 1.2 g·L^−1^; K_2_SO_4_ – 14.9 g·L^−1^; KOH – 18.2 g·L^−1^; NaCl – 4.13 g·L^−1^; glycerol – 40 g·L^−1^; 85% H_3_PO_4_ – 27 mL·L^−1^). After the fed‐batch, the MeOH induction was started by injection of 5 g MeOH directly into the fermenter and afterwards by a stepwise increase in MeOH feed from 0 to 6 g·h^−1^ over 2 h. Glycerol feed was reduced from 15 to 6 g·h^−1^ over 2 h and a mixed MeOH/glycerol feed was maintained until the end of the fermentation. In total, 400–500 g MeOH was fed to the cultures before harvest after 96 h of induced growth. Samples were taken for western blot analysis at different time points. The cell pellet was collected by centrifugation (2704 ***g***, 30 min, 4 °C) and stored at −20 °C.

### Enzyme purification

Cell lysates were prepared by Merckenschlager (Braun Biotech International, Melsungen, Germany) glass beads cell homogenization under CO_2_ cooling (3‐min disruption steps). For every 1 g harvested wet cell pellet, 2‐mL cell lysis buffer (50 mm HEPES/NaOH, 150 mm NaCl, 35 mm imidazole, 1 mm PMSF, pH 7.8) and a spatula tip of FAD were added before cell disruption. The lysates were cleared by centrifugation (38 720 ***g***, 30 min, 4 °C) and filtered through a paper filter. Nickel ion affinity chromatography was done by applying the lysates onto self‐packed nickel‐Sepharose 6 Fast Flow columns (GE Healthcare) at 4 °C. Afterwards, the material was washed with washing buffer (50 mm HEPES/NaOH, 150 mm NaCl, 75 mm imidazole, pH 7.8) and the enzyme stripped with elution buffer (50 mm HEPES/NaOH, 150 mm NaCl, 300 mm imidazole, pH 7.8). The purification was monitored by SDS/PAGE and fractions containing hDMGDH were concentrated and rebuffered to storage buffer (50 mm HEPES/NaOH, 150 mm NaCl, pH 7.8) using Amicon ultracentrifugal filter units (50 kDa cut‐off; Merck‐Milipore, Darmstadt, Germany). The obtained enzyme purity was sufficient for kinetic and photometric studies of the enzymes. If a higher enzyme purity was needed, especially for protein crystallization, the eluted fractions of the affinity chromatography were rebuffered to buffer A (25 mm HEPES/NaOH, 25 mm NaCl, pH 7.8), and anion exchange chromatography with a MonoQ 5/50 GL column (GE Healthcare) was done on an ÄKTA FPLC system (GE Healthcare) at 4 °C. The protein was eluted with a gradient from 0% to 50% buffer B (25 mm HEPES/NaOH, 1 m NaCl, pH 7.8). The chromatography was controlled by SDS/PAGE and fractions containing hDMGDH were concentrated and rebuffered to storage buffer as described above. Purification was done identically for the wild‐type and the variant enzyme.

### SDS/PAGE

Enzyme samples were separated by SDS/PAGE, with 12.5% separation gels (for western blot 10%), under reducing conditions as described by Laemmli 35. Gels were either used for western blot analysis or stained with Coomassie brilliant blue R‐250 for purification control. As protein standard, a PageRuler Prestained protein ladder (Thermo Fisher Scientific) was employed.

### Western blot

*K. phaffii* cell lysates for western blot screening and control of fermentation were prepared by glass bead disruption following the Invitrogen EasySelect^™^ *Pichia* Expression Kit manual (p. 42, Thermo Fisher Scientific). Western blot analysis was done on nitrocellulose membranes essentially following the General Protocol for western blotting from Bio‐Rad (Hercules, CA, USA; Bulletin 6376). As antibodies, a rabbit anti‐histidine IgG antibody at 1 : 2000 overnight at 4 °C as primary and an HRP‐linked goat anti‐rabbit IgG antibody at 1 : 5000 for 1 h at room temperature as secondary antibody were used. As loading control, a rabbit anti‐GAPDH antibody was used in a 1 : 1000 dilution overnight. Immunoreactive signals were obtained using SuperSignal West Pico Chemiluminescent Substrate (Thermo Fisher Scientific) and detection in a G:BOX (Syngene, Cambridge, UK).

### UV‐Vis absorption spectroscopy

UV‐Vis absorption spectra to assess protein concentration, activity, purity, quality, and cofactor saturation, as well as for steady‐state kinetic measurements and photoreduction were recorded with a Specord 210 spectrophotometer (Analytik Jena, Jena, Germany).

### Enzyme quantification and calculation of the extinction coefficient

Protein concentrations of purified hDMGDH enzymes were determined according to the characteristic absorption of bound FAD at 450 nm. The molar extinction coefficient for hDMGDH was calculated using the method described by Macheroux 36 to 11 600 m ^−1^·cm^−1^.

### Steady‐state kinetics

Steady‐state kinetic parameters were determined using DCPIP according to Okamura‐Ikeda *et al*. 37 or ferrocenium hexafluorophosphate (Fc^+^ ${\text{PF}}_{6}^{-}$) as described by Lehman *et al*. 38 as terminal electron acceptor and measurable dimension in the spectrophotometer. In short, DCPIP assays were performed at 25 °C and pH 7.8 in 50 mm HEPES/NaOH, 150 mm NaCl, 135 μm DCPIP, and 100 nm hDMGDH with freshly prepared 3 mm phenazine methosulfate (PMS) as intermediate electron mediator and 0–100 mm dimethylglycine (DMG) following the change of absorption spectrophotometrically at 600 nm over 3 min. Ferrocenium assays were performed at 25 °C at pH 7.8 in 50 mm HEPES/NaOH, 150 mm NaCl, 0.1 mm EDTA, 100 nm hDMGDH, 0–100 mm DMG, and freshly prepared 0.2 mm Fc^+^ ${\text{PF}}_{6}^{-}$ following the change of absorption at 300 nm over 3 min. For each concentration, at least a triplicate measurement was performed, the initial velocities were determined, and *K*_M_ and *k*_cat_ were assessed by nonlinear hyperbolic fit in origin 8.6 (OriginLab Corp., Northampton, MA, USA).

### Presteady‐state kinetics

Presteady‐state reaction kinetics were measured anaerobically with a Hi‐Tech stopped flow instrument (SF‐61DX2; TgK Scientific Limited, Bradford‐on‐Avon, UK) in a glove box (Belle Technology, Weymouth, UK) at 25 °C. For determination of the reductive rates of enzyme‐bound FAD, 20 μm purified hDMGDH in 50 mm HEPES/NaOH, pH 7.8 containing 150 mm NaCl was shot against different concentrations of DMG (0.25–250 mm) or sarcosine (0–500 mm) dissolved in 50 mm HEPES/NaOH, 150 mm NaCl pH 7.8 and monitored at 460 nm with a KinetaScanT diode array detector (MG‐6560; TgK Scientific Limited). Each concentration was measured in triplicates and the observed rate constants (*k*_obs_) for different substrate concentrations were calculated using an exponential fitting function in the kinetic studio software (TgK Scientific Limited). The dissociation constant (*K*_D_) was determined by a hyperbolic fitting curve employing origin 8.6 (OriginLab Corp.). The oxidative rate (*k*_ox_) of the enzyme was measured three times at an oxygen concentration of 10.5% O_2_ (135 μm O_2_), by mixing substrate‐reduced hDMGDH with air equilibrated buffer (50 mm HEPES/NaOH, 150 mm NaCl, pH 7.8).

### Determination of the redox potential

The redox potential (*E*_0_) of the enzyme‐bound FAD was measured by the dye‐equilibration method using the xanthine/xanthine oxidase system as described by Massey 27. The concentrations of enzyme and redox dye were chosen in a way that their absorption maxima were in the same range. The reactions were performed with a Hi‐Tech stopped flow device (SF‐61DX2; TgK Scientific Limited). The measurements took place under anaerobic conditions in a glove box (Belle Technology) at 25 °C in 50 mm HEPES/NaOH, 150 mm NaCl, pH 7.0. The simultaneous reduction of FAD and the redox dye was monitored with a KinetaScanT diode array detector (MG‐6560; TgK Scientific Limited). The reaction was started by mixing a solution containing 300 μm xanthine, 5 μm benzyl viologen, and an appropriate amount of enzyme with a solution containing catalytic amounts of xanthine oxidase (approximately 200 nm, from bovine milk, Grade III purity; Sigma‐Aldrich) and the redox dye indigotrisulfonic acid dipotassium salt (*E*_0_, pH 7.0, 25 °C = −81 mV). The redox potential was calculated using double logarithmic plots, log(ox/red) of the enzyme versus log(ox/red) of the dye, according to Minnaert 39. A linear least‐squares fit was done with Excel 2010 (Microsoft, Redmond, WA, USA).

### Anaerobic photoreduction and reoxidation

Photoreduction of hDMGDH was done according to the procedure reported by Massey and Hemmerich 40. About 20 μm purified enzyme sample in 50 mm HEPES/NaOH, pH 7.8 containing 150 mm NaCl, 1 mm EDTA, 1 μm 5‐diaza‐FMN, and 2 μm methyl viologen were rendered anaerobic by 2‐h incubation in sealable quartz cuvettes in a glove box (Belle Technology). Photoirradiation was carried out with a 10 W LED flood light (Luminea, Buggingen, Germany) and cooling of the cuvette to 15 °C. Spectra were recorded until no further changes were observed. For reoxidation of the enzyme, the cuvettes were exposed to air oxygen and absorption spectra were recorded until no further changes were observed.

### Temperature stability

#### Thermofluor^®^

The temperature stability of the enzymes was assessed by monitoring the change in fluorescence using the solvatochromic dye SYPRO Orange (Sigma‐Aldrich) in a Thermofluor^®^ assay. Thermofluor^®^ measurements were carried out as reviewed elsewhere 41 in an FX Connect real‐time PCR system (Bio‐Rad) in 25 μL of 50 mm HEPES/NaOH, pH 7.8, containing 150 mm NaCl, 10 μm of enzyme, and 2.5 μL of a 500× dilution of a SYPRO Orange Enzyme Gel stain. The samples were preheated to 25 °C and then the temperature was increased in 0.5 °C·min^−1^ steps to 95 °C. Fluorescence data were collected using the FRET channel. Melting temperatures (*T*_m_) were determined using cfx manager 3.0 software (Bio‐Rad).

#### Circular dichroism spectroscopy

CD spectra were recorded on a PS‐150J spectropolarimeter (Jasco, Groß‐Umstadt, Germany) using a 0.02 cm water‐jacketed cylindrical cell thermostatically controlled by an external computer‐controlled water bath (Julabo F25, Seelbach, Germany). Thermal denaturation data were recorded in a temperature range from 25 to 95 °C with a heating rate of 1 °C·min^−1^, a response of 1 s, and a resolution of 0.1 °C. The protein concentration used was approximately 6 μm in 50 mm HEPES/NaOH, 50 mm NaCl, pH 7.8. The melting temperature was estimated as the point of inflection of the temperature‐dependent mean residue ellipticity at 208 nm.

### Crystallization of hDMGDH

Crystallization experiments were performed with the microbatch method using different commercial crystallization screens [Index and Morpheus Screen from Hampton Research (Aliso Viejo, CA, USA) and Molecular Dimensions (Newmarket, UK), respectively]. Drops were prepared by mixing 0.5 μL of the protein solution (at a concentration of 4 mg·mL^−1^ in 25 mm HEPES, 25 mm NaCl, and 2 mm DTT, pH 7.5) with an equal volume of mother liquor using an ORYX 8 pipetting robot (Douglas Instruments, Hungerford, UK). The drops were sealed with a 3 : 1 mixture of paraffin and silicon oil and the trays incubated at 20 °C. First crystal clusters were observed after approximately 1 month in various conditions. Further optimization involved dilutions of the original condition using 25 mm HEPES, 25 mm NaCl, 2 mm DTT, pH 7.5. Diffracting hDMGDH crystals were obtained with a 1 : 3 dilution of the original condition H5 of the Morpheus Screen consisting of 10% w/v PEG 20000, 20% v/v PEG MME 550, 0.02 m of each amino acid, and 0.1 m MOPS/HEPES‐Na pH 7.5.

### Data collection and processing

X‐ray diffraction data were collected to a maximum resolution of 3.1 Å on beam line ID29 (λ = 0.972 Å) at the ESRF Grenoble, France. The crystals were monoclinic (space group *P*2_1_) with unit‐cell parameters *a* = 83.38 Å, *b* = 119.87 Å, *c* = 86.47 Å, and β = 92.6°. The data were processed using the xds package 42 and the structure was solved by molecular replacement using the structure of the rat dimethylglycine dehydrogenase (PDB ID: 4PAA, 90% sequence identity) 18. Structure rebuilding and refinement were performed using the programs coot 43 and phenix 44. The model contained one B‐factor per amino acid residue. Noncrystallographic‐symmetry (NCS) restraints were applied. A small number of water molecules were added only manually into the most significant electron difference electron density peaks before the last cycle of refinement. Clear electron density was observed for the majority of the amino acids except of the first 18 N‐terminal residues and the 13 C‐terminal residues in both chains present in the asymmetric unit. Residual density was interpreted as FAD in both chains. The final structure was validated using molprobity 45. Detailed statistics pertaining to data processing and structure refinement are summarized in Table 2. The atomic coordinates and structure factors have been deposited in the Protein Data Bank under the accession number 5L46.

### *In silico* procedures

Figures for structural analyses were prepared using pymol (DeLano Scientific, San Carlos, CA, USA 46). *In silico* mutagenesis of hDMGDH residue H109 to R109 in the crystal structure was performed with the mutagenesis wizard of pymol (DeLano Scientific) and subsequent energy minimization using yasara (Yasara Biosciences, Vienna, Austria). Docking experiments of DMG and sarcosine into the hDMGDH structure was done using autodock/vina 47 as implemented in yasara. Cavity analyses after and before mutagenesis were performed with the LIGSITE algorithm (CASoX plugin of pymol, 48).

## Author contributions

PA has expressed, purified, and characterized the wild‐type enzyme and the variant; AH, TPK, and KG crystallized proteins and determined the crystal structure of WT hDMGDH; PA and PM designed biochemical experiments and interpreted the data; PA, AH, TPK, KG, and PM wrote the manuscript.

## Conflict of interests

The authors declare no conflict of interests.
